# Supplementary material for: Identification of Freezing-Responsive microRNAs and Their Targets in Chinese Jujube by Small RNA and Degradome Sequencing
Source: Int J Mol Sci. 2026 Sep 4;27(17):7912. doi: 10.3390/ijms27177912 (PMC13566890; doi:10.3390/ijms27177912)
Supplement: Supplementary file 1 [file ijms-27-07912-s001.zip › Supplementary Material S2/novel_miR_40.pdf]

Provisional ID : novel_miR_40
Score total : 0.6
Score for star read(s) : -1.3
Score for read counts : 0
Score for mfe : 0.3
Score for randfold : 1.6
Score for cons. seed :
Total read count : 56
Mature read count : 56
Loop read count : 0
Star read count : 0

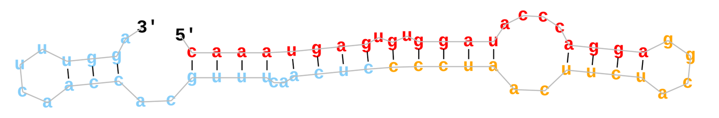

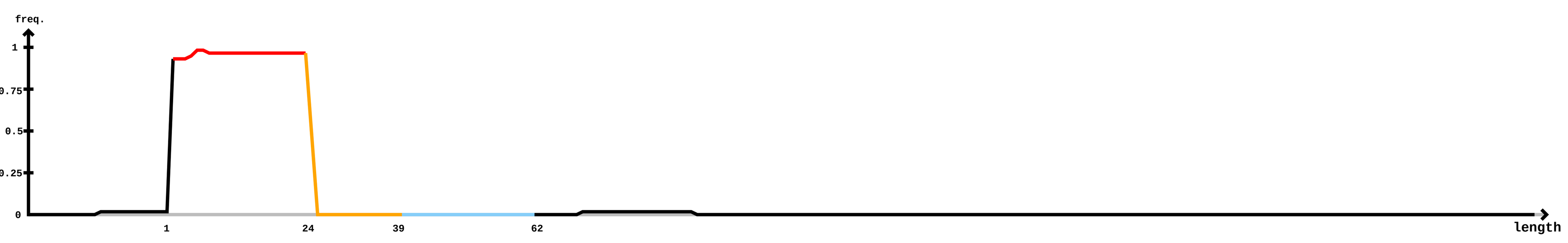

Mature   Star

```
5'- uucaagcaauacagcuugccuccaaaugaguguggauacccaggaggcaucuucaaucccccucaacuuugcaccaacuuuggaggccaaggaaaaggagcucgcaaaugguaauaaucuuaaaaaauacaaacuacaauuuauaaauuugaaaauuuugcuaugaugggcaucuggcuuuugauuacaugaaauucggauaauacacuagacauccauaauaaacaagccgagguugccuaucauagcag -3'  exp
    ..(((((......)))))(((((((((.(((((((.....(((.((.((.....)))))))....)))))))))..))))))))((........))......(((((.((((............................))))..)))))......(((((((((((((((((((((((...((((.........((((............)))).))))....))))).))).)))))))))))))))  reads  mm  sample
    ..........acagcuugccuccaaaug..............................................................................................................................................................................................................................  1  0  LT-1
    ......................caaaugaguguggauacccagga.............................................................................................................................................................................................................  30  0  LCK-2
    ......................caaNugaguguggauacccagga.............................................................................................................................................................................................................  1  1  LCK-1
    ......................caaaugaguguggauacccagga.............................................................................................................................................................................................................  21  0  LCK-1
    ......................caaaugaguguggCuacccagga.............................................................................................................................................................................................................  1  1  LCK-1
    .........................augaguguggauacccagga.............................................................................................................................................................................................................  1  0  LT-2
    ..........................ugaguguggauacccagga.............................................................................................................................................................................................................  1  0  JT-2
    ..........................ugaguguggauacccagga.............................................................................................................................................................................................................  1  0  JT-3
    ..........................................................................................gaaaaggagcucgcaaaug.............................................................................................................................................  1  0  JCK-1
```
